# Performances of Cement Mortar Incorporating Superabsorbent Polymer (SAP) Using Different Dosing Methods

**DOI:** 10.3390/ma12101619

**Published:** 2019-05-17

**Authors:** Yawen Tan, Huaxin Chen, Zhendi Wang, Cheng Xue, Rui He

**Affiliations:** 1 School of Materials Science and Engineering, Chang’an University, Xi’an 710061, Shanxi, China; tywen1210@126.com (Y.T.); hxchen@chd.edu.cn (H.C.); 2 State Key Laboratory of Green Building Materials, China Building Materials Academy, Beijing 100024, China; wzhendi@163.com; 3 CCCC Second Highway Engineering CO., LTD, Xi’an 710065, Shaanxi, China; xuecheng3a@163.com

**Keywords:** superabsorbent polymer (SAP), compressive strength, drying shrinkage, freeze–thaw resistance

## Abstract

Modified cement mortar was prepared by incorporating a superabsorbent polymer (SAP) with two kinds of dosing state, dry powdery SAP and swelled SAP (where the SAP has been pre-wetted in tap water), respectively. The mechanical properties, drying shrinkage and freeze–thaw resistance of the mortars were compared and analyzed with the variation of SAP content and entrained water-to-cement ratios. Additionally, the effect of SAP on the microstructure of mortar was characterized by scanning electron microscopy (SEM). The results indicate that agglomerative accumulation is formed in the voids of mortar after water desorption from SAP and there are abundant hydration products, most of which are C-S-H gels, around the SAP voids. The incorporation of the powdery SAP increases the 28 d compressive strength of the mortars by about 10% to 50%, while for the incorporation of swelled SAP, the 28 d compressive strength of the mortar can be increased by about −26% to 6%. At a dosage of 0.1% SAP and an entrained water–cement ratio of 0.06, the powdery SAP and the swelled SAP can reduce the mortar shrinkage rate by about 32.2% and 14.5%, respectively. Both the incorporation of powdery and swelled SAP has a positive effect on the freeze–thaw resistance of cement mortar. In particular, for powdery SAP with an entrained water-to-cement ratio of 0.06, the mass loss rate after 300 cycles is still lower than 5%.

## 1. Introduction

Superabsorbent polymers (SAPs) are excellent high-performance liquid absorbent and retention polymers, which can absorb and retain several hundred times higher amounts of water or aqueous liquids relative to their own mass in a short time due to their three-dimensional hydrophilic network structures [1,2,3]. Taking advantage of such specific characteristics, SAP has been widely applied in many fields [4,5,6,7], such as concrete, agriculture, personal hygiene, waste treatment, and medical industries [8,9,10]. In concrete in particular, the typical application of SAP is placed on developing internal curing technology, which refers to storing an amount of water in the interior of cement during the mixing process of the cementitious materials by adding internal curing materials [11,12].

SAP is often introduced to cement-based materials as a promising new internal curing material for concrete [13,14]. When SAP is exposed to cement-based mixtures, a transition in the osmotic pressure gradient and chemical potential occurs between the inside and outside of the three-dimensional network leading to water absorption and storage of the SAP [15,16,17]. The stored water can be released to compensate for the internal moisture loss of cement-based materials, which can promote further hydration of the cement particles and reduce self-desiccation shrinkage during the hydration of cement or evaporation of the water [18,19]. Therefore, the addition of SAP can significantly mitigate shrinkage in low water-to-cement ratio mixtures and greatly reduce the occurrence of cracking [20]. Additionally, SAP can also increase the degree of hydration, enhance the freeze–thaw resistance capacity, change the rheological behaviors and optimize the pore size distribution [21,22,23], among other actions.

However, the majority of recent studies about SAP have focused on the effect and theory on the shrinkage, hydration and mechanical performances of cement-based materials due to the variation of SAP content and entrained water-to-cement ratios [24,25,26]. There are relatively few studies devoted to the influence of dosing methods of SAP and the majority of the studies choose to add dry SAP powder to other components of the cement-based materials for mixing [27,28,29]. By now, the popular dosing methods of SAP can be classified as dry powdery SAP and swelled SAP (where SAP has been pre-wetted in tap water), which correspond to the dry powder and swelled gel form, respectively, when adding them to the cement-based materials.

When SAP powder is added to cement-based mixtures, although it shows a favorable dispersibility in cement-based materials, a common phenomenon of “competitive absorption” is prone to occur between SAP and cement-based materials during mixing and remarkably influences the workability and rheology of mixtures [30,31,32]. As for the swelled SAP, it normally releases the water too rapidly in cement-based mixtures, which can increase the total water-to-cement ratio, impair the effectiveness of internal curing, and work against the mechanical performance of cement-based materials [33,34,35].

In addition, the swelled SAP is generally difficult to disperse uniformly, which can result in the partial internal curing of cement-based mixtures. That said, the dosing methods of SAP can have a significant impact on the water kinetics of SAP during cement hydration and the mechanical performance and durability of cement-based materials. Therefore, the issue of adequate preparation and the adding method of SAP into the concrete is crucial for the fulfillment of internal curing. The current research aims to investigate the appropriate dosing method of SAP (dry powdery SAP or swelled SAP) and provide the benefits of the hydration of cement-based materials.

In this research, two different adding methods were designed to prepare the cement mortar for concrete internal curing. The mechanical properties, shrinkage properties and freeze–thaw resistance of cement mortar were compared and analyzed with the variation of SAP content and entrained water-to-cement ratio. The effect of SAP on the microstructure and pore structure of concrete was characterized by scanning electron microscopy (SEM).

## 2. Materials and Methods

### 2.1. Materials

The cement used in this study was P.I 42.5 (from China United Cement Corporation, Beijing, China) and the chemical composition can be found in Table 1. The Chinese ISO standard was used. The superplasticizer was a polycarboxylate-based aqueous solution with a solid content of 35%.

**Figure 1:**
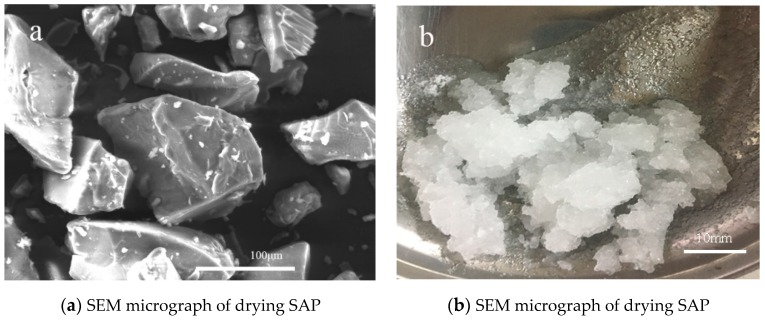
The superabsorbent polymer (SAP) particle characteristics: scanning electron microscopy (SEM) micrograph (**a**) and swelled image (**b**).

The SAP, a copolymer of acrylamide and sodium acrylate, was used in cement mortars. The particle diameters of SAP ranged from 80 μm to 200 μm, and the absorption capacities of the de-ionized water and cement filtrate solution with a swelling time of 5 min were 150.0 ± 2.6 g/g and 30.5 ± 0.9 g/g, respectively [23,34]. Tap water was used as mixing and internal curing water. The particle shape, size and surface characteristics of SAP were observed by SEM, as shown in Figure 1a. The swelled SAP is shown in Figure 1b.

### 2.2. Mixture Preparation and Testing Methods

#### 2.2.1. Two Adding Methods of Superabsorbent Polymer (SAP)

**Figure 2:**
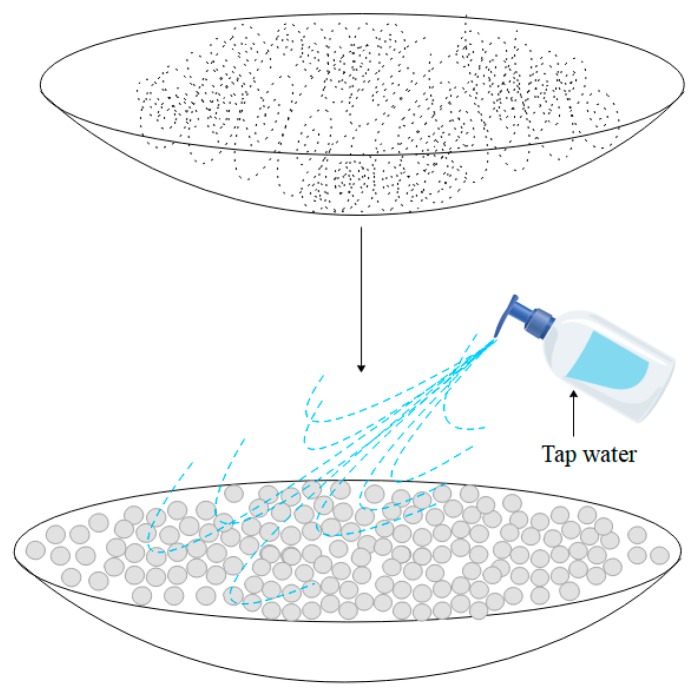
Preparation process of swelled SAP.

Two adding methods with powder and swelled SAP, respectively, were designed in cement-based materials. One was where the powder of SAP was added directly, and the other was described as the detailed procedure in Figure 2. Firstly, the particles of SAP were uniformly paved on a watch glass that was slightly larger than the SAP coverage area (SAP thickness layer was less than 0.2 mm), and then a given volume of water (the amount of water was closely related to SAP content and entrained water-to-cement ratios), which was poured in an injector and uniformly sprayed on the particles of SAP to make each particle of SAP fully swelled. Finally, the swelled SAP was added into other dry components of a concrete mix.

#### 2.2.2. Preparation and Testing of Internal Curing Mortar

The mix composition of mortar is shown in Table 2. Two reference cement mortars with a water-to-cement ratio (W/C) of 0.42 and 0.48, respectively, were designed. The dosage of sand was 2 times that of cement. For the consistence of fresh mixture, superplasticizer was added in an amount of 0.5 wt% and 0.2 wt% (mass-% of cement weight), respectively, to ensure practical workability for the 0.42 and 0.48 mixtures, which were denoted as Ref-1 and Ref-2, respectively. The amount of superplasticizer was adjusted to ensure practical fluidity (210 ± 10 mm).

Cement mortar with a water-to-cement ratio of 0.42 is prone to self-shrinkage. Based on the model of Powers and Brownyard [13], the amount of entrained water needed in the SAP can be calculated and amounts to an entrained water-to-cement ratio (w/c)_e_ of 0.06 [16]. Therefore, the effective water-to-cement ratio of cement mortar is 0.42 (w/c)_ref_ and the total water-to-cement ratio is 0.48 (w/c)_tot_. Furthermore, an amount of about 0.2% SAP can also be calculated based on the absorption capacity of the cement filtrate solution in Section 2.1.

**Figure 3:**
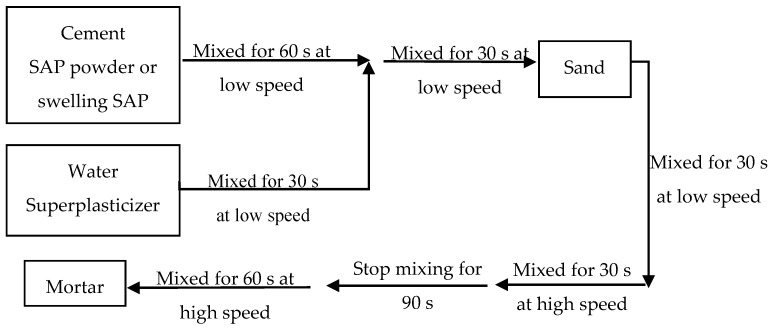
Mixing procedure of cement mortar with SAP.

The cement mortars containing 0.1%, 0.2%,0.3%, and 0.4% (mass-% of cement weight) SAP in the form of powders and which did not have an additional water-to-cement ratio, were denoted as P_0.1%_-0, P_0.2%_-0, P_0.3%_-0, and P_0.4%_-0, respectively. The cement mortars P_0.1%_-0.06, P_0.2%_-0.06, P_0.3%_-0.06, and P_0.4%_-0.06 respectively represented the cement mortars with the powders of 0.1%, 0.2%, 0.3%, and 0.4% (mass-% of cement weight) SAP and an additional water-to-cement ratio of 0.06 (w/c)_e_. Likewise, the mortars S_0.1%_-0.06, S_0.2%_-0.06, S_0.3%_-0.06, and S_0.4%_-0.06, respectively, represented the cement mortars with the 0.1%, 0.2%, 0.3%, and 0.4% (mass-% of cement weight) swelled SAPs and an additional water-to-cement ratio of 0.06(w/c)_e_. In addition, when the cement mortars contained 0.3% SAP, the additional water-to-cement ratio was considered from 0.02 to 0.08 to investigate the effects of the additional water-to-cement ratio with different adding methods, and which were denoted as P_0.3%_-0.02, P_0.3%_-0.04, P_0.3%_-0.08, S_0.3%_-0.02, S_0.3%_-0.04, and S_0.3%_-0.08, respectively. The amount of superplasticizer in SAP cement mortars was the same as for Ref-1, in order to decrease the influence on the setting time [16]. The mixing program of raw materials is shown in Figure 3.

Specimens with the shape of 160 × 40 × 40 mm^3^ were prepared to study the compressive strength. Three specimens were prepared for each mortar for each of the performed tests. The specimens molded were kept in the environment of 20 °C and 98% humidity for 24 h, and were then demolded and cured under the conditions for 27 days. The strength compressive testing was carried out according to the Chinese standard (GB 17671), and the specimens were tested with a controlled load rate of 2.4 ± 0.2 kN/s.

Specimens with a size of 25 × 25 × 280 mm^3^ were used to evaluate the drying shrinkage, and the testing process of shrinkage specimens was performed in accordance with the Chinese standard (JC/T 603). The self-desiccation shrinkage of the specimens was considered to be mainly generated during the first three days [32]. Therefore, the deformation after three days was recorded as the drying shrinkage of the specimens in the paper. The drying shrinkage specimens molded were also kept in the environment of 20 °C and 98% humidity for 24 h, and were then demolded and cured under water for 2 d. Following this, they were removed from the water, the surface water was wiped off with a damp cloth and a comparator with an accuracy of 0.01 mm was used to measure the initial value (*l*_0_). Finally, the specimens were moved to a room of 20 °C and 60% ± 5% humidity until the desired ages (i.e., 7 d, 14 d, 38 d and 35 d) to measure the data (*l*_i_) for that age. The drying shrinkage ratio can be calculated according to Equation (1).

s = (*l*_i_ − *l*_0_)/250 × 100 (1)

Specimens with a size of 160 × 40 × 40 mm^3^ were used to explore the freeze–thaw resistance performance. Furthermore, six specimens were prepared for each mortar for each of the performed tests. All specimens molded were kept in the environment of 20 °C and 98% humidity for 24 h, and were then demolded and cured under the conditions for 23 days. Following this, specimens were cured in water for another four days. Finally, specimens aged 28 days were used for the freezing and thawing test. The specific procedure of the freeze–thaw test was performed in accordance with the Chinese standard (GB/T 50082-2009). The mass and dynamic elasticity modulus were tested once after an interval of 25 times cycles, and the mass loss and relative dynamic elastic modulus were calculated. Every freeze–thaw cycle continued for 3.5 h, with the highest temperature of 18 ± 2 °C and lowest temperature of 20 ± 2 °C. Furthermore, the standard requirements are that the freeze-thaw test can be stopped when it reaches any of the following three conditions:

① The number of freeze–thaw cycles reaches 300;
② The relative dynamic elastic modulus of the specimen is less than 60% of the initial value;
③ The rate of mass loss of the specimen is more than 5%.

## 3. Results and Discussion

### 3.1. The Microstructure around SAP Voids

No matter how the SAP was added into the mixture, given SAP voids, were reserved in the hardened mortar after losing the stored water of powdery or swelled SAP. The SAP voids are closely correlated with the pore structure, strength, drying shrinkage, and permeability of cement mortars [20,32]. Therefore, it is necessary to observe the void characteristics reserved from SAP.

**Figure 4:**
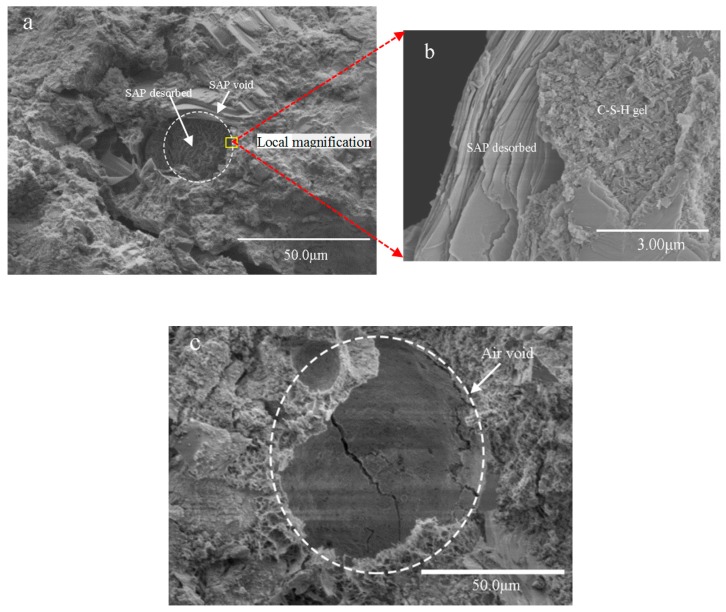
SEM images of cement paste with the addition of powdery SAP at 28 days: (**a**) microscopic structure of the SAP void; (**b**) microscopic interface between the SAP and hardened mortar; (**c**) microscopic structure of the air void.

The microscopic structure of P_0.3%_-0.06 pores is shown in Figure 4. It can be observed from Figure 4a that the shape of the SAP void is irregular and the outer diameter is approximately 30–40 μm. It can also be observed that the SAP pore diameter is smaller than the SAP particle size mentioned in Section 2.1, which may be due to the filling or covering of the partial SAP voids by the hydration product. The formation of the large SAP void has some adverse effects on the compressive strength of the specimen [25]. However, SAP particles can be curled easily and agglomerated to form an agglomerative accumulation after the desorption of water in the mortar [32]. Therefore, SAP voids in mortar are completely different from normal air voids (Figure 4c). Figure 4b shows the observed interface between the SAP and hardened matrix. It can be seen from Figure 4b that there are abundant hydration products, especially C-S-H gels, around the SAP void, which is different from the loose hydration products around the normal air void (Figure 4c).

### 3.2. Mechanical Properties

**Figure 5:**
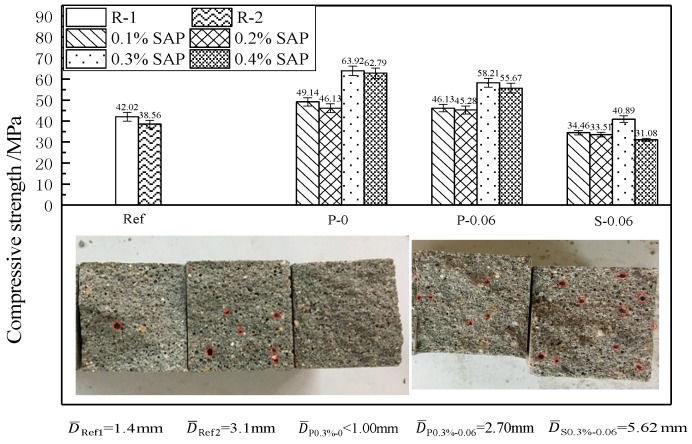
Compressive strength of cement mortar with different adding methods of SAP.

**Figure 6:**
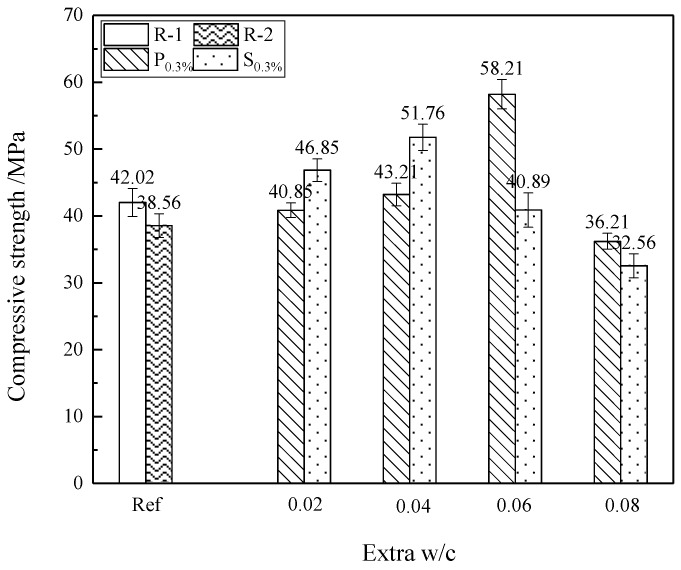
Compressive strength of cement mortar with different entrained water-to-cement ratio (w/c) values.

It is known that many efforts have been devoted to studying the mechanical properties of cement-based materials with SAP, while the influence of SAP addition on the compressive strength of cement-based materials is still controversial. The addition of SAP introduces a certain amount of SAP voids into the cement-based materials, which may decrease the mechanical properties of cement-based materials reported in the majority of existing literature [25,36]. Conversely, the other studies thought that SAP did not negatively affect the mechanical properties of cement-based materials due to the increased hydration of cement [37]. The 28 d compressive strength of cement mortars which had been designed by the corresponding adding methods of SAP and entrained w/c were evaluated, and the results are shown in Figure 5 and Figure 6. In addition, Figure 5 also shows the effect of SAP on the macroscopic pores visible to the human eye, which was measured using a Vernier caliper with an accuracy of 0.02 mm on the fracture surface of mortar after the end of the compressive strength test.

As expected, the reference mortar with w/c = 0.48 shows a lower compressive strength than that with w/c = 0.42 at the age of 28 d, shown in Figure 5. Furthermore, compared with the reference mortars (R-1 and R-2), the incorporation of the powdery SAP raises the 28 d compressive strength of the mortars by about 10% to 50%, while for the incorporation of swelled SAP, the 28 d compressive strength of the mortar can be increased by about −26% to 6%. It indicates that the 28 d compressive strength of the mortars is closely related to the dosing method of SAP, and the incorporation of powdery SAP is more contributed to the development of the 28 d compressive strength of mortar than that of swelled SAP. This can be attributed to the “competitive water absorption” between the SAP and cemented materials when SAP is added to the mortar in powder form [30,32].

Compared with other dosages of SAP, the mortar with 0.3% SAP has the highest compressive strength for the two adding methods (Figure 5). Thus, the macroscopic pore characteristic in the broken section of the specimens, for which 0.3% SAP and 0.06 (w/c)_e_ were added, was studied specifically. The average size of the visible pores in the broken section of the specimen (P_0.3%_-0 and P_0.3%_-0.06) containing powdery SAP is smaller than that containing swelled SAP, and the average pore size of the mortar without additional w/c (P_0.3%_-0) is always less than 1mm. However, the average size of mortar with swelled SAP (S_0.3%_-0.06) is 5.62 mm, which may result in a significant reduction in the compressive strength of the mortar.

In addition, it can also be observed from Figure 6 that the compressive strength of P_0.3%_ at an additional water–cement ratio of 0.06 for 28 d is 58.21 MPa, whereas the compressive strengths of R-2 and S_0.3%_ with the same total water-to-cement ratio (w/c = 0.48) are 38.56 MPa and 40.89 MPa, which are 0.66 and 0.7 times the of compressive strength of P_0.3%_ respectively. Furthermore, the mortar containing 0.3% powdery SAP and an entrained water-to-cement ratio of 0.06 exhibits a higher compressive strength than the Ref_1_, Ref_2_ and other mortars containing SAP. It may be explained that an appropriate entrained w/c can positively promote the potential of internal curing for the further hydration of cement-based materials, as well as refine the voids and improve the mechanical properties of the mortar.

### 3.3. Drying Shrinkage

**Figure 7:**
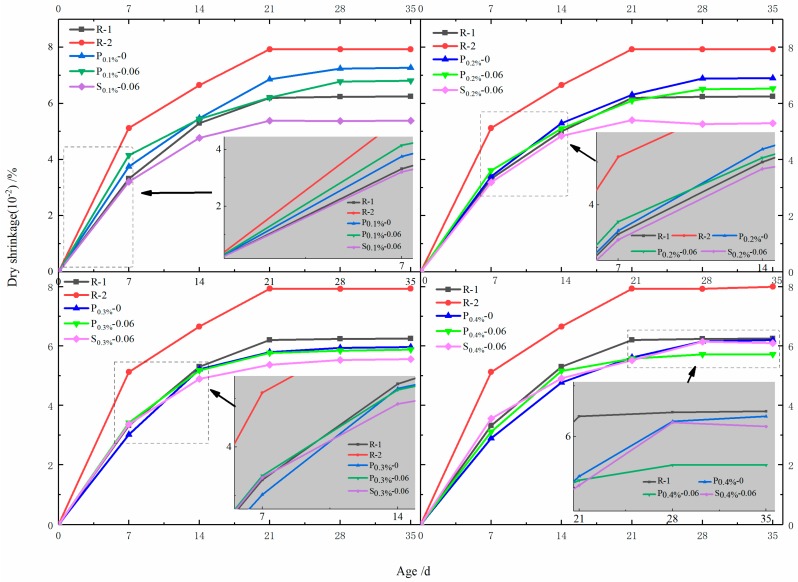
Drying shrinkage of cement mortar with different adding methods of SAP.

**Figure 8:**
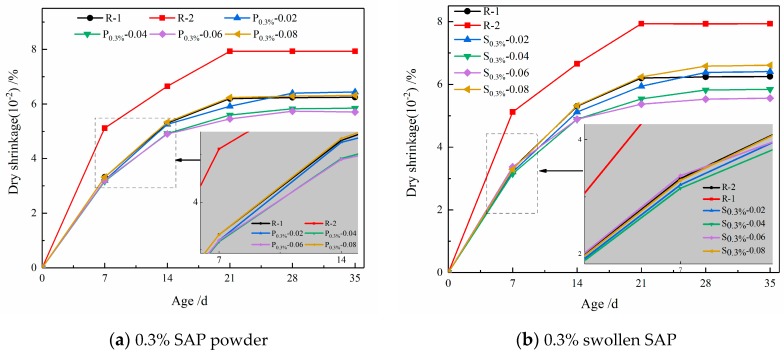
Drying shrinkage of cement mortar with different entrained w/c values.

Dry shrinkage is a type of reduction in the volume that is due to the loss of moisture in hardened cement mortar. Therefore, it is well established that moisture in hardened cement mortar is of great significance to dry shrinkage [38]. The results of drying shrinkage of cement mortars with SAP are presented in Figure 7 and Figure 8.

Figure 7 shows that for the specified four different dosages of SAP, the rate of shrinkage greatly varies before 21 days, and then slightly increases. As existing studies have reported [39,40], the drying shrinkage is closely related to the total water–cement ratio and generally raises with the increase of the total water-to-cement ratio. Therefore, the reference mortar with w/c = 0.48 shows obviously higher drying shrinkage than that with w/c = 0.42 during the testing process. Noticeably, the rate of drying shrinkage is significantly affected by the methods of adding SAP. The drying shrinkage of R-2 at a dosage of 0.1% for 28 d is 7.93 × 10^−^^4^, whereas the drying shrinkage of P_0.1%_-0.06 and S_0.1%_-0.06 with the same total water–cement ratio (w/c = 0.48) are only 5.38 × 10^−^^4^ and 6.78 × 10^−^^4^, which reduced the drying shrinkage rate by about 32.2% and 14.5%, respectively. Furthermore, the rate of drying shrinkage with swelled SAP was even lower than that of R-1 (w/c = 0.48) throughout the test. It can be seen that, when the moisture in the pores of mortar decreased due to evaporation and hydration, the water reserved in SAP could release and re-fill the pores to alleviate the surface tension [41]. In addition, the water stored by swelled SAP could release water more easily and earlier to re-fill the pores than that of the powdery SAP, and thus the reduction of drying shrinkage caused by swelled SAP was much more obvious.

It can be also seen from Figure 7 that, despite the adding methods, compared to 0.1% and 0.2% SAP content, both 0.3% and 0.4% contents show a more evident reduction in drying shrinkage of cement mortars. Additionally, the drying shrinkage of cement mortars that added 0.3% and 0.4% SAP was lower than those without SAP (R-1 and R-2) after 14 days. Furthermore, compared with 0.4% SAP, the dosage of 0.3% SAP can better reduce the drying shrinkage of the mortar, which indicates that the drying shrinkage of mortar is also closely related to the amount of swelled SAP.

Figure 8a,b depict the effect of the entrained w/c on the drying shrinkage of mortar for a given 0.3% powder state and swelled SAP, respectively. It can be found that the entrained w/c is closely related to the drying shrinkage of mortar [39]. It can be seen from Figure 8a that the drying shrinkage of R-1 at a dosage of 0.3% for 28 d is 6.24 × 10^−^^4^, whereas the drying shrinkage of P_0.3%_-0.02, P_0.3%_-0.04, P_0.3%_-0.06, and P_0.3%_-0.08 with the same effective water- cement ratio (w/c = 0.42) are 6.4 × 10^−^^4^, 5.83 × 10^−^^4^, 5.74 × 10^−^^4^, and 6.29 × 10^−^^4^, respectively. Therefore, when the entrained w/c is within the range of 0.04 to 0.06, the drying shrinkage can be decreased with the addition of powdery SAP; furthermore, a similar result can be found in Figure 8b. However, when the entrained w/c is 0.08, the effect of powdery and swelled SAP on reducing dry shrinkage was obviously decreased during the whole test process compared with the entrained water-to- cement ratios of 0.02, 0.04, and 0.06. It can be explained by the fact that, too much entrained water increases the water–cement ratio of mortar, which can facilitate the drying shrinkage of mortar.

### 3.4. Freeze–Thaw Resistance

**Figure 9:**
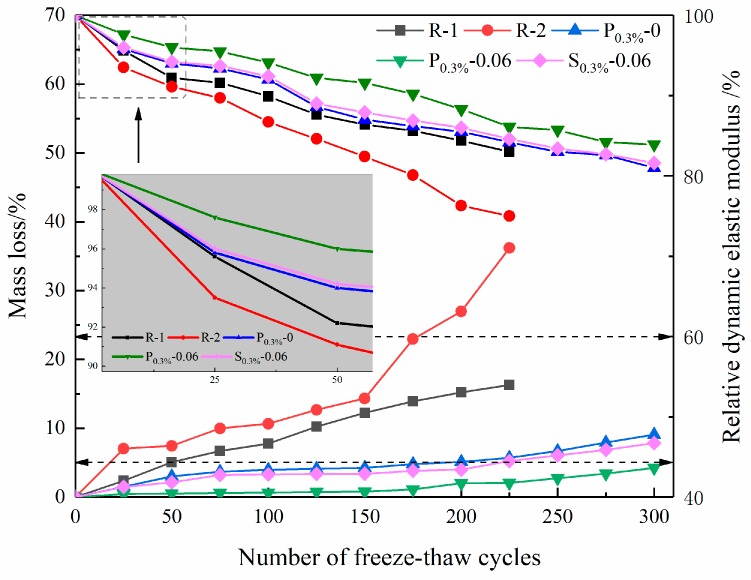
Mass loss and relative dynamic elastic modulus of cement mortar with different adding methods of SAP in freeze-thaw cycles.

It can be observed that if the dosage of powdery and swelled SAP and the entrained water-to-cement ratio in the mortar are 0.3% and 0.06, respectively, the cement mortar exhibits good mechanical properties (Section 3.2) and drying shrinkage resistance (Section 3.3). Consequently, for a given entrained water-to-cement ratio of 0.06 and 0.3% SAP, the freeze-thaw resistance of the mortar was investigated by measuring the mass loss rate and relative dynamic elastic modulus of the mortar under different freeze-thaw cycles in Figure 9.

It can be found from Figure 9 that the mass losses of R-1 and R-2 are 16.3% and 36.2% after 225 freeze–thaw cycles, whereas the mass losses of P_0.3%_-0, P_0.3%_-0.06 and S_0.3%_-0.06 are 5.7%, 2.1%, and 5.2%, respectively. Therefore, the introduction of SAP can improve the freeze–thaw resistance of the mortar, especially the powdery SAP. Similar findings can also be observed in the relative dynamic elastic modulus curves. It can be explained by the fact that, when the dosage of SAP and the entrained water-to-cement ratio are selected properly, the introduction of SAP may introduce a number of SAP voids in the cement mortar, which has a positive impact on the frost resistance of cement mortar. Furthermore, the addition of SAP can increase the air content of the fresh mortar [42]. However, the scaling takes place on the surface rather than on the inside of the specimen during the freeze–thaw cycles. Compared with powdery SAP, swelled SAP is prone to release water prematurely during mixing, and some of the released water could perspire on the surface of the specimen. Therefore, the mass loss of the mortar containing the swelled SAP increases during the freeze-thaw cycles and the strength drops obviously.

## 4. Conclusions

The performances of cement mortar incorporating SAP by different adding methods were studied. Based on the results presented, the following conclusions can be drawn:

(1) SAP voids in mortar are completely different from normal air voids and SAP particles can be easily curled and agglomerated to form an agglomerative accumulation after the desorption of water in the mortar. There are abundant hydration products around SAP voids, especially C-S-H gels;
(2) Compared with the reference mortars (R-1 and R-2), the incorporation of the powdery SAP increases the 28 d compressive strength of the mortars by about 10% to 50%, while for the incorporation of swelled SAP, the 28 d compressive strength of the mortar can be increased by about −26% to 6%;
(3) At a dosage of 0.1% SAP and an entrained water–cement ratio of 0.06, the powdery SAP and the swelled SAP can reduce the mortar shrinkage rate by about 32.2% and 14.5%, respectively. When the entrained w/c is within the range of 0.02 to 0.06, the drying shrinkage decreased with the increase of entrained w/c for the two adding methods of SAP;
(4) The addition of SAP can improve the freeze–thaw resistance of the mortar, especially for the mortar with the powdery SAP and an entrained water-to-cement ratio of 0.06, and the mass loss rate after 300 cycles is still lower than 5%.

In the concrete industry, the primary merit of SAP is to reduce the shrinkage of cement-based materials. Thus, a further research effort is needed to characterize the influence of dry powdery SAP and swelled SAP (where the SAP has been pre-wetted in tap water) on the self-shrinkage and chemical shrinkage of cement-based materials.

## Figures and Tables

**Table 1:** Chemical and mineral composition of cement.

Chemical Compositions	CaO	SiO_2_	A1_2_O_3_	Fe_2_O_3_	MgO	Na_2_O	SO_3_
Mass percentage (%)	65.36	22.15	4.51	3.39	2.31	0.49	0.46
Mineral compositions	C_3_S	C_2_S	C_2_A	C_4_AF			
Mass percentage (%)	56.54	20.87	6.22	10.31			

**Table 2:** Design of SAP-modified cement mortars.

Code	Cement/g	Sand/g	Effective w/c	Entrained w/c	Total w/c	SAP
R-1	495	990	0.42	0	0.42	0
R-2	495	990	0.48	0	0.48	0
P_0.1%_-0	495	990	0.42	0	0.42	0.10%
P_0.2%_-0	495	990	0.42	0	0.42	0.20%
P_0.3%_-0	495	990	0.42	0	0.42	0.30%
P_0.4%_-0	495	990	0.42	0	0.42	0.40%
P_0.1%_-0.06	495	990	0.42	0.06	0.48	0.10%
P_0.2%_-0.06	495	990	0.42	0.06	0.48	0.20%
P_0.3%_-0.06	495	990	0.42	0.06	0.48	0.30%
P_0.4%_-0.06	495	990	0.42	0.06	0.48	0.40%
S_0.1%_-0.06	495	990	0.42	0.06	0.48	0.10%
S_0.2%_-0.06	495	990	0.42	0.06	0.48	0.20%
S_0.3%_-0.06	495	990	0.42	0.06	0.48	0.30%
S_0.4%_-0.06	495	990	0.42	0.06	0.48	0.40%
P_0.3%_-0.02	495	990	0.42	0.02	0.44	0.30%
P_0.3%_-0.04	495	990	0.42	0.04	0.46	0.30%
P_0.3%_-0.08	495	990	0.42	0.08	0.50	0.30%
S_0.3%_-0.02	495	990	0.42	0.02	0.44	0.30%
S_0.3%_-0.04	495	990	0.42	0.04	0.46	0.30%
S_0.3%_-0.08	495	990	0.42	0.08	0.50	0.30%
